# The effects of integrating work-related factors and improving cooperation in musculoskeletal physical therapy practice: protocol for the ‘WORK TO BE DONE’ cluster randomised controlled trial

**DOI:** 10.1186/s12891-020-03375-2

**Published:** 2020-06-08

**Authors:** Nathan Hutting, Wiebke Oswald, Maria W.G. Nijhuis - van der Sanden, Monique Filart, Tamara Raaijmakers, Hendrik J. Bieleman, J. Bart Staal, Yvonne F. Heerkens

**Affiliations:** 1 School of Organisation and Development, Research Group Occupation & Health, HAN University of Applied Sciences, P.O. Box 6960, 6503 GL Nijmegen, The Netherlands; 2 School of Allied Health, Physical Therapy, HAN University of Applied Sciences, Nijmegen, The Netherlands; 3 Radboud Institute for Health Sciences, IQ healthcare, Radboud university medical centre, Nijmegen, The Netherlands; 4 School of Health, Physical Therapy, Saxion University of Applied Sciences, Enschede, The Netherlands; 5 Saxion University of Applied Sciences, Research Group Health and Physical Activity, Enschede, The Netherlands; 6 Centre Work Health, Amersfoort, The Netherlands; 7 School of Allied Health, Musculoskeletal Research Group, HAN University of Applied Sciences, Key factors in Physiotherapy and Allied Health Research Group, Nijmegen, The Netherlands

**Keywords:** Musculoskeletal disorders, Physical therapy, Occupational health, Cluster randomised trial

## Abstract

**Background:**

Musculoskeletal disorders (MSDs) are the primary cause of disability worldwide and a major societal burden. Recent qualitative research found that although a patient’s work is considered important, physical therapists take work participation insufficiently into account as a determining factor in the treatment of patients with MSDs. Therefore, the aim of this study is to improve the effectiveness of physical therapy (in primary healthcare) with respect to the work participation of employees with MSDs by increasing the knowledge and skills of generalist physical therapists and by improving the collaboration between generalist physical therapists and physical therapists specialised in occupational health.

**Methods/design:**

This trial is a two-arm non-blinded cluster randomised controlled trial. Working patients with MSDs visiting a physical therapy practice are the target group. The control group will receive normal physical therapy treatment. The intervention group will receive treatment from a physical therapist with more knowledge about work-related factors and skills in terms of integrating work participation into the patients’ care. Data are gathered at baseline (T0), at four months (T1) and eight months (T2) follow-up. Most outcomes will be assessed with validated patient-reported questionnaires. Primary outcomes are the limitations in specific work-related activities and pain during work. Secondary outcomes include limitations in general work-related activities, general pain, quality of life, presenteeism, sick leave (absenteeism), estimated risk for future work disability, work-related psychosocial risk factors, job performance, and work ability. Based on a sample size calculation we need to include 221 patients in each arm (442 in total). During data analysis, each outcome variable will be analysed independently at T1 and at T2 as a dependent variable using the study group as an independent variable. In addition to the quantitative evaluation, a process evaluation will be performed by interviewing physical therapists as well as patients.

**Discussion:**

The trial is expected to result in a more effective physical therapy process for working patients with MSDs. This will lead to a substantial reduction of costs: lower costs thanks to a more effective physical therapy process and lower costs due to less or shorter sick leave and decreased presenteeism.

**Trial registration:**

Netherlands Trial Register, registration number: NL8518, date of registration 9 April 2020, URL registration: https://www.trialregister.nl/trial/8518

## Background

Musculoskeletal disorders (MSDs) are the primary cause of disability worldwide and a major societal burden [1]. MSDs are characterised by pain and reduced physical function, often associated with increased risk of developing other chronic health conditions, increased all-cause mortality, limitations in daily activities, restricted participation, and a significant decline in mental health decline and quality of life [2, 3]. Moreover, MSDs are associated with long-term disability that is often resistant to current treatments [4]. Work-related MSDs are disorders whereby work-related activities and conditions significantly contribute to the onset or progression of the disorder, but are not necessarily the sole cause of the disorder [5]. Whether work-related or not, musculoskeletal complaints can have a significant impact on work in terms of reduced productivity, sickness absence and long-term incapacity to work [6].

Musculoskeletal health is critical to human functioning, enabling mobility, dexterity, and the ability to work and actively participate in all aspects of life. Musculoskeletal health is therefore essential in maintaining human capital as well as economic, social and functional independence across the life course [7]. Work is associated with positive benefits, including both mental and physical health [8]. Social factors such as work, employment and economic status are important health determinants [9], and ‘having a job’ reflects an individual’s ability for functioning as a part of their overall health status [10]. Moreover, the longer individuals are out of work due to MSDs, the harder it is for them to get back to work [11]; early intervention is therefore advocated [12]. In addition, long-term work absence poses a serious risk to physical, mental and social wellbeing, while return to work can improve recovery for individuals with common health problems [8]. Therefore, early discussions about work with individuals are crucial in order to avoid lengthy sick leave which results in fewer treatment gains and greater costs [13].

Although most health professionals, including physical therapists, acknowledge the importance of their patients’ work, occupation and the ability to work, these topics are often not addressed within regular Dutch healthcare [14–18]. Recent qualitative research in the Netherlands found that although a patient’s work is considered important, physical therapists take work participation insufficiently into account as a determining factor in the treatment of patients with MSDs. They often lack specific knowledge about work-related factors, and there is insufficient cooperation between generalist physical therapists and other occupational healthcare providers (including physical therapists specialised in occupational health, occupational therapists and exercise therapists) [18].

In a survey of Dutch physical therapists, 64% of the 142 respondents indicated that occupational factors should be addressed to a greater extent within physical therapy. Only 14.8% of the respondents indicated that they communicate with or consult a physical therapist specialised in occupational health. Only 12.7% of the participants who do not have a specialised physical therapist within their practice sometimes/regularly refer patients to a specialised physical therapist [17]. The participating physical therapists stated that if they communicate with or consult other occupational health professionals, they mainly have contact with occupational health/insurance physicians (72.5%) and occupational therapists (31.7%).

These issues were also seen in qualitative research conducted among general practitioners in the Netherlands which found that general practitioners seemed well aware of the relationship between work and health but needed more knowledge, communication skills and better cooperation with occupational physicians to manage work-related problems. Participants reported that they lacked the knowledge to advise patients specifically concerning their work environment [16].

To redress this imbalance, it is important that healthcare becomes more work-focused [19, 20]. Health professionals need to formulate goals related to work participation, give suitable consideration to work outcomes and manage chronic health conditions to optimise functional capacity [21]. There is robust evidence to suggest that a lack of work-focused healthcare (i.e. the failure of health professionals to address work-related issues in the clinical encounter) is an obstacle to work participation [20]. Work-focused healthcare involves healthcare providers taking an interest in, and accepting responsibility for, addressing obstacles to work participation in the clinical encounter [22].

Important elements in the integration of occupational health into primary healthcare include training primary healthcare professionals to recognise early work-related ill health, to provide advice on improving working conditions and health at work, to support return to work, and to preserve and restore work capacity [21]. Healthcare professionals, including physical therapists, need to take into account patients’ work-related difficulties and their own perceived ability to offer effective guidance, and consider the ‘receptivity’ of employment contexts to patients’ work problems, in order to ensure a smooth transition back to work [14]. Therefore, gaining an understanding of the relationship between health and work should be part of the training of all healthcare professionals [21].

As far as we know, no studies have been conducted in primary healthcare that investigate the effects of work-focused physical therapy for working patients with MSDs. The aim of this study is to improve the effectiveness of physical therapy (in primary healthcare) with respect to the work participation of employees with MSDs by increasing the knowledge and skills of generalist physical therapists and by improving the collaboration between generalist physical therapists and physical therapists specialised in occupational health. The study will consider the following research question: To what extent will integrating work-related factors into the care processes of generalist physical therapists and improving cooperation between generalist physical therapists and physical therapists specialised in occupational health, enhance the effectiveness of physical therapy (in primary healthcare) for working patients with MSDs?

Our hypothesis is that the intervention will result in an increased knowledge of work participation and the relevant factors that influence work participation, which will make it easier to integrate work participation into the care regimen of generalist physical therapists and to decide when referral to or consulting with a physical therapist specialised in occupational health is appropriate. This will improve the effectiveness of care, leading to faster recovery (pain and limitations in activities) and a higher quality of life for patients with MSDs. This also means fewer sessions and reduced recurrences, thus decreasing the costs of healthcare and the costs due to absenteeism and presenteeism.

## Methods/design

### Trial design

**Fig. 1:**
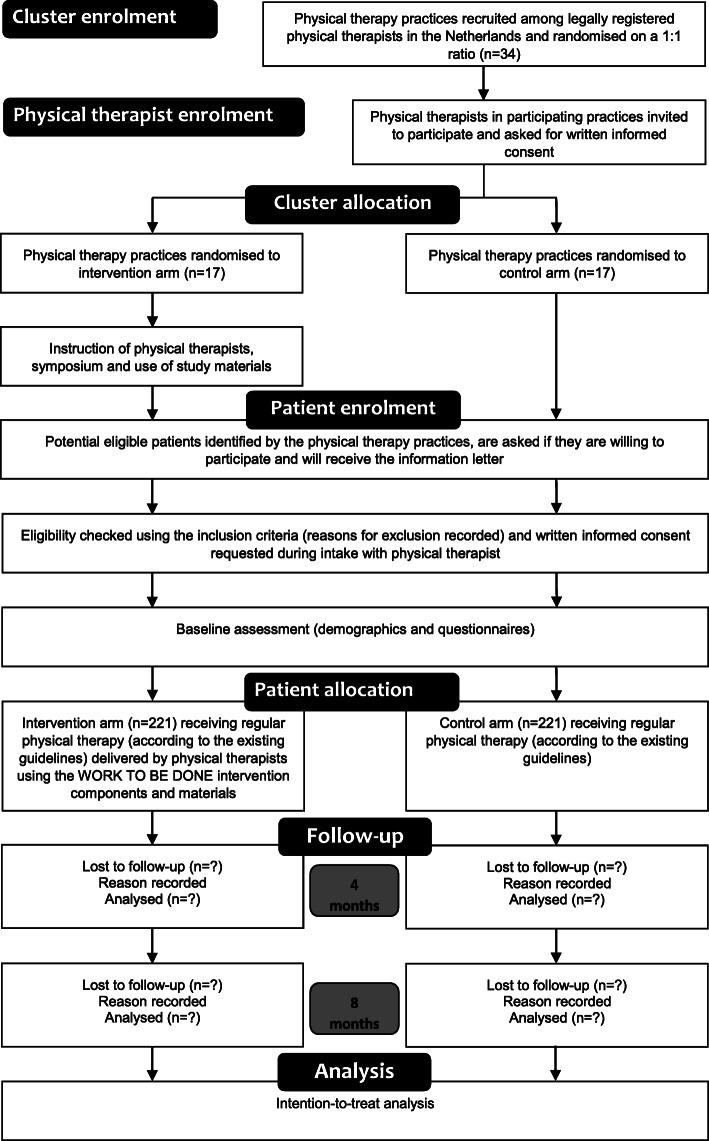
Trial phases and participant flow

This trial is a two-arm non-blinded cluster randomised controlled trial (CRCT). Outcomes are assessed at baseline and at four and eight months after baseline. This protocol complies with the SPIRIT guidelines [23]. The trial will be reported in accordance with the CONSORT guidelines [24]. For the stakeholders involved, this study is entitled: ‘WORK TO BE DONE: integrating work participation into shared decision-making in physical therapy practice’. The short title of the intervention is ‘WORK TO BE DONE’ (in Dutch: WERK AAN DE WINKEL). Figure 1 shows the trial phases and participant flow. Members of the project group (NH, WO, JBS, YH) participated in the design of the trial and the intervention and will participate in each stage of the trial. The advisory group consisting of the relevant stakeholders (see Acknowledgements) provided input on the design of the intervention and will be consulted for advice throughout the trial.

### Study setting

This is a Dutch CRCT (Trial registration: Netherlands Trial Register: NL8518). The protocol of the study and data management plan have been uploaded to Open Science Framework (https://doi.org/10.17605/OSF.IO/KDUYS). The trial will be conducted in physical therapy practices in the Netherlands. Participating physical therapy practices will be the unit of randomisation (cluster). Working patients with MSDs visiting a physical therapy practice are the target group. The control group will receive regular physical therapy treatment. The intervention group will receive treatment from a physical therapist with more knowledge about work-related factors and more skills in terms of integrating work participation into the patients’ care.

### Participants and recruitment

Participating physical therapy practices will be recruited from among legally registered physical therapists in the Netherlands. Physical therapists in the recruited physical therapy practices can participate in this study if they treat patients with MSDs and do not have a recognised specialisation in the area of occupational physical therapy or work-related physical therapy. Physical therapists will be recruited via announcements made by the stakeholders involved in newsletters and on websites, and via social media.

Patients will be recruited by the participating physical therapists. In order to be eligible for participation in this study, a patient must meet all of the following criteria:

1. Display one or more musculoskeletal complaints
2. Have an indication for physical therapy treatment
3. Have an employment contract or be self-employed (normally working ≥12 h a week)
4. Experience symptoms during work or in their own opinion have problems performing their work (including absenteeism).

Patients who are unable to access and fill in the online follow-up questionnaires will be excluded.

### Randomisation and blinding

The physical therapy practices of the participating physical therapists will be the unit of randomisation (cluster). Each practice will be randomly assigned to either the intervention or control arm on a 1:1 basis. The allocation sequence will be generated online (with a block size of six) [25] using http://randomization.com by the principal investigator. Participating practices will be randomised in the order that they confirm their participation in the study. The practices will be informed about the group allocation by the principal investigator. Based on the randomisation, physical therapists working in the randomised practices will be able to use all the ‘WORK TO BE DONE’ intervention components (intervention group) or will continue providing regular physical therapy to their patients (control group). Physical therapists and patients cannot be blinded to allocation group.

### Procedures

As soon as a physical therapy practice confirms participation in this study, they will receive an email from the principal investigator indicating to which group they have been allocated. All participating physical therapists will receive patient information about the study and materials needed for the inclusion of patients. Patients will be recruited and informed by the participating physical therapy practices. Consecutive patients will be asked if they are willing to participate in this study and will receive the information letter. Patients will be allowed to use all the time they need to consider their participation. As soon as a patient has decided to participate, the first consult will be planned. Patients will also receive an information letter and will be given the time they need to consider their participation. If patients do not want to participate in the study, they will receive regular physical therapy treatment and will not be enrolled in the study. If patients want to participate in the study, the physical therapist will assess them for eligibility during the intake and ask for written informed consent and their contact details. The physical therapist will then send the contact details to the investigators via a secured app (Siilo, Siilo Holding B.V., the Netherlands). An image of the informed consent form will also be sent to the investigators via a secured app. The original form will be sent by regular mail or will be collected by the investigators.

### Interventions

#### Development

The intervention content is based on earlier published research of the authors [17, 18] and qualitative research conducted within the development process of this study. This qualitative research consisted of focus groups with generalist physical therapists, occupational therapists and exercise therapists (total participants = 16); focus groups with physical therapists, occupational therapists and exercise therapists specialised in occupational health, and with other relevant healthcare providers involved in occupational health (total participants = 22); and focus groups with patients from the target population of the intervention (total participants = 18). The results of these focus group studies will be published elsewhere. The intervention was developed by the first two authors, in cooperation with the other authors of this publication. All members of the advisory group had the opportunity to comment on the development and content of the intervention.

#### Control group

Physical therapy practices randomised to the control group will provide regular physical therapy (according to the existing guidelines) to their patients. These patients will be asked to participate in the study and to fill in the baseline and follow-up questionnaires.

#### Intervention group

Physical therapy practices randomised to the intervention group will provide regular physical therapy (according to the existing guidelines). In addition, they are able to use all the ‘WORK TO BE DONE’ intervention components and materials. These invention components and materials are:

##### Symposium

At the start of the intervention, a full-day symposium will be held with presentations about the importance of work-focused healthcare, information about the trial and collaborating with other occupational health professionals. There will also be a three-hour masterclass about shared decision-making. The symposium will be video recorded for physical therapists who are unable to attend the symposium.

##### E-learning

Physical therapists must follow an e-learning course consisting of two parts. The first part contains general information about the importance of work-focused healthcare, the interaction between work and health, and (work-related) factors influencing participation in work. The second part contains more specific information and guidance about addressing patients’ work participation in the diagnostic and treatment phase and about working with occupational health professionals, including guidance on cooperation between generalist physical therapists and physical therapists specialised in occupational health.

##### Online toolkit

Physical therapists can use an online toolkit to easily find information about providing work-focused care. Using the keyword search functionality, they can find information about laws and regulations, assessment and other tools, questionnaires, and occupational health professionals. Moreover, the toolkit contains short information about all the topics covered in the e-learning course.

##### Network

Physical therapists will be part of a local network through which they can easily contact occupational health physical therapists, exercise therapists specialised in occupational health, and occupational therapists with additional training in occupational health.

##### Patient information

Physical therapists can use patient information developed by the authors highlighting the importance of work-focused healthcare.

#### Co-interventions

No restrictions with regard to co-interventions will be set. Both the intervention group and the control group will be allowed to use all other interventions (co-interventions). We will ask for participation in co-interventions in the follow-up measurements.

### Outcome assessment and data collection

All outcome measures will be self-reported measures. Data will be collected using online questionnaires filled in by the participating patients at the start of the intervention (T0), four months after the start of the intervention (T1, short-term effects) and eight months after the start of the intervention (T2, long-term effects). All questionnaires will be developed using Qualtrics online survey software (Qualtrics®). Outcome measures will be collected from the patients. The schedule for enrolment, outcome measures and time points is summarised in Table 1. Physical therapists will be asked to fill in a questionnaire at the start of the intervention and after eight months.

**Table 1:**
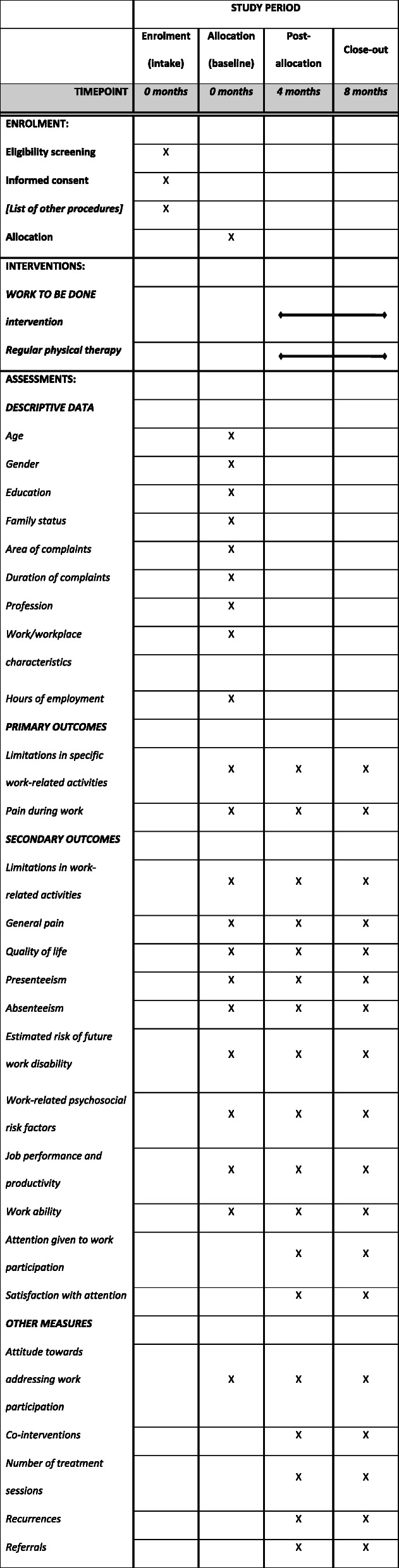
Schedule for enrolment, outcome measures and time points.

#### Descriptive data

Characteristics of the patients will be collected at baseline in the first questionnaire. Patient characteristics include age, gender, education, family status, area of complaints, duration of complaints, profession and work/workplace, and hours of employment.

Characteristics of the physical therapists will be collected at baseline with the first questionnaire. Physical therapist characteristics include age, gender, education, area of specialisation and years of experience as a physical therapist.

#### Primary outcomes

##### Limitations in specific work-related activities

The limitations in specific work-related activities in the previous week will be assessed using a patient-specific functional scale (PSFS) [26, 27]. Patients will be asked to identify the most important work-related activity they are unable to perform or are having difficulty with as a result of their musculoskeletal problems. Patients will be asked to rate each activity on an 11-point scale indicating the current level of difficulty associated with each activity. The PSFS is a valid, reliable and responsive outcome measure for patients with MSDs [26, 28–30]. As the minimal clinically important difference (MCID) we will use two points [31–34].

##### Pain during work

The level of pain experienced by the patient in the previous week during work will be assessed using the 11-point numeric pain rating scale (NPRS). The NPRS has been established as reliable and valid [32, 35–37]. The MCID of the NPRS in patients with musculoskeletal complaints is two points [32–34].

#### Secondary outcomes

**Limitations in general work-related activities** will be assessed using a single question about the limitations experienced during work in general due to the complaints (11-point scale) [38].

**General pain**. The general level of pain experienced by the patient in the previous week will be assessed using the 11-point numeric pain rating scale (NPRS).

**Quality of life** will be assessed using the 12-item Short-Form Health Survey (SF-12) [39].

**Presenteeism** will be assessed using the Dutch version of the 6-item Stanford Presenteeism Scale (SPS-6) [40].

**Absenteeism** will be measured by asking the patient the number of days they had been out of work due to their complaints during the previous month.

**Estimated risk for future work disability** will be assessed using the Örebro Musculoskeletal Pain Screening Questionnaire (short form) [41].

**Work-related psychosocial risk factors** will be assessed using the blue flags questionnaire [42].

The degree to which health problems interfere with specific aspects of **job performance** and the **productivity impact** of these work limitations will be assessed using the Work Limitations Questionnaire [43].

**Work ability** will be assessed using the Work Ability Index-Single Item Scale (WAS), which is a responsive measure for work participation and highly predictive for future sickness absence [44].

The amount of **attention to work participation** given by the physical therapist, and the **level of satisfaction** of the patient with this attention will be measured using five-point Likert scales.

#### Other measures include

In addition, we will collect data on the attitude towards addressing work participation in physical therapy practice (five-point Likert scale), the use of other healthcare interventions (co-interventions), the number of physical therapy treatment sessions, recurrences of complaints and referrals to other occupational health professionals (including physical therapists specialised in occupational health). All participating physical therapists (in the intervention arm as well as the control arm) will receive a questionnaire about their awareness, attitude, knowledge, and self-efficacy with regard to the treatment of working patients with MSDs at T0 and T2. Information about their work experience with the intervention and the use of the intervention materials will be collected at T2 (intervention group only).

### Sample size

The sample size was calculated based on the main outcome measures (11-item NPRS and 11-point PSFS), an expected effect size of 0.7 and a standard deviation of 1.8 [32, 45–47]. On the basis of two-sided testing, significance level of 0.05, power 0.8, accounting for the cluster design effect, assuming an ICC coefficient of 0.05, and 15 clusters per intervention arm, an effective sample size would require 10 patients per cluster (i.e. per physical therapy practice). With respect to the effect of cluster size variation, we added 10% additional clusters (in total 17 clusters per arm) [48]. Allowing for a 25% loss to follow-up, we would need to recruit 13 participants (i.e. patients) per cluster [49]. In total, we need to include 221 patients in each arm (442 in total).

### Data analyses

Analysis of the two groups will be conducted blinded to the treatment allocation and the data will be analysed according to the intention-to-treat principle. Baseline characteristics of the participants will be presented in means and standard deviations (symmetrically distributed continuous variables), median interquartile ranges (other continuous variables), and counts and percentages (categorical variables), and will be checked for baseline differences between the two groups.

All outcome measurements will be continuous variables and will be presented as means and standard deviations. Normality of the data will be checked and verified using histograms, normal probability plots and Shapiro-Wilk tests. Between-group differences for all outcomes will be analysed using linear regression. If data appears to be not normally distributed, Mann-Whitney U tests or log transformation will be performed.

Each outcome variable will be analysed independently at T1 and at T2 [50] as a dependent variable using the study group as an independent variable, adjusted for the baseline measurement of each outcome measure. Adjustment for confounding will only be applied if the regression coefficient of the intervention variable changes by more than 10% when the potential confounding variable is added to the model. If missing data for an outcome is > 5%, multiple imputation will be conducted. Results will be considered significant if p < 0.05. All analyses will be performed using IBM SPSS Statistics 26 (IBM Corporation).

### Process evaluation

In addition to the quantitative evaluation of outcomes, a process evaluation will be performed by interviewing (up until the point of data saturation) 7–12 physical therapists (directly after the inclusion period has ended) of the intervention group to learn more about their experiences with the newly developed method and the additional value of the network. In addition, (up until the point of data saturation) 7–12 patients in the intervention group will be interviewed (2–3 months after the start of the intervention) to learn about their experiences with the approach and their own role in the recovery process. All the physical therapists of the intervention group will also receive a questionnaire about their experiences with the intervention (descriptive data) at T2.

### Ethics

The Research Ethics Committee of the Radboud university medical centre reviewed the study protocol and has declared (declaration no. 2018–4465) that the study does not fall within the remit of the Medical Research Involving Human Subjects Act (WMO) in the Netherlands and can be carried out (in the Netherlands). Because the study does not fall within the remit of the WMO, no data monitoring committee is mandatory. The research will be carried out in compliance with the Declaration of Helsinki on Ethical Principles for Medical Research Involving Human Subjects. Confidentiality is guaranteed and participants will receive information about the purpose and processes of the study. If they so wish, participants can withdraw from the study at any time, for any reason, without the need for an explanation and without any consequences. No restrictions with regard to other treatments will be placed on participants. The sponsor and funder will have no influence on the study design; collection, management, analysis, and interpretation of data; writing of the report; and the decision to submit the report for publication.

### Confidentiality

All data collected will be regarded as confidential. Paper formats will be stored in a locked closet in a locked room. All online data will be gathered using software dedicated to protecting all data based on industry best practices. All data will be stored using the facilities of the HAN University of Applied Sciences in accordance with current guidelines. Only the principal investigator and co-investigator will have access to this anonymized database.

### Safety

The newly developed intervention is expected to have no potential threats for the patients. Participants will only be asked to fill in the questionnaires and some of them will be interviewed about their experiences. According to Dutch regulations, the study does not fall within the remit of the Medical Research Involving Human Subjects Act (WMO). Therefore, in accordance with Dutch regulations, no data monitoring committee is necessary. The principal investigator and leading investigator will meet at least every three weeks to monitor adverse events, any issues relating to the trial and to review the recruitment and trial progress.

### Dissemination of study results

When the trial is completed, the method and the products can be implemented nationwide within physical therapy practices. The patient information will be freely available for all patients. The online toolkit and the e-learning programme can be used by all members of the Royal Dutch Society for Physical Therapy (KNGF). We will keep the online toolkit running and up to date for at least three years after the end of the trial. Given that the lecturers of the HAN and Saxion physical therapy bachelor’s educational programmes’ are directly involved in the trial, the results of the trial can be integrated directly into these study programmes.

The results of the study will be published in international and Dutch peer-reviewed journals and professional journals, and will be presented at national and international conferences. The results will also be disseminated through the information channels of the project and advisory group members.

## Discussion

This CRCT will investigate to what extent integrating work-related factors in the care processes of generalist physical therapists and improving the cooperation between generalist physical therapists and physical therapists specialised in occupational health will enhance the effectiveness of physical therapy (in primary healthcare) for working patients with MSDs. Our hypothesis is that the intervention will result in better patient health and an increase in the knowledge of generalist physical therapists regarding work participation and the relevant factors that influence work participation, which will make it easier for them to integrate work participation into care and to decide when referral to or consulting with a physical therapist specialised in occupational health or another occupational health professional is appropriate.

After participating in this trial, generalist physical therapists delivering the ´WORK TO BE DONE` intervention will have increased their knowledge and will have integrated work-related factors in a structural and process-oriented way into their care processes. They will be better able to treat work-related and work-relevant complaints, resulting in less overtreatment. In addition, overtreatment will be reduced as generalist physical therapists will refer patients at an earlier stage to a physical therapist specialised in occupational health or another occupational health professional within the network.

For the patients, the trial is expected to result in faster recovery (pain and limitations in activities) and a higher quality of life. In addition, we expect that patients will have less absenteeism and be less limited in work-related activities.

The trial is expected to result in a more effective physical therapy process for working patients with MSDs. This will mean a substantial reduction of costs: lower costs thanks to a more effective physical therapy process and lower costs due to less or shorter sick leave and lower presenteeism. A process evaluation will be carried out which will provide insights in the facilitators and barriers with regard to implementation of the intervention.

## Data Availability

Not applicable.

## Abbreviations

MSDs: Musculoskeletal disorders
CRCT: Cluster randomised controlled trial
MCID: Minimal clinically important difference
PSFS: Patient-specific functional scale
NPRS: Numeric pain rating scale
WMO: Medical Research Involving Human Subjects Act
